# Assessment of imaging risks for recurrence after stereotactic radiosurgery for brain metastases (IRRaS-BM)

**DOI:** 10.1186/s12885-024-12636-5

**Published:** 2024-07-18

**Authors:** Yun Hwa Roh, Ji Eun Park, Seo Young Park, Young Hyun Cho, Young-Hoon Kim, Sang Woo Song, Shinkyo Yoon, Ho Sung Kim

**Affiliations:** 1 Department of Radiology and Research Institute of Radiology, University of Ulsan College of Medicine, Asan Medical Center, Seoul, Republic of Korea; 2 Department of Statistics and Data Science, Korea National Open University, Seoul, Republic of Korea; 3 Department of Neurosurgery, University of Ulsan College of Medicine, Asan Medical Center, Seoul, Republic of Korea; 4 Department of Oncology, University of Ulsan College of Medicine, Asan Medical Center, Seoul, Republic of Korea; 5 Department of Radiology, Samsung Medical Center, Sungkyunkwan University School of Medicine, Seoul, Republic of Korea

**Keywords:** MRI, Brain metastasis, Stereotactic radiosurgery, Tumor habitat analysis, Recurrence

## Abstract

**Background:**

The identification of viable tumors and radiation necrosis after stereotactic radiosurgery (SRS) is crucial for patient management. Tumor habitat analysis involving the grouping of similar voxels can identify subregions that share common biology and enable the depiction of areas of tumor recurrence and treatment-induced change. This study aims to validate an imaging biomarker for tumor recurrence after SRS for brain metastasis by conducting tumor habitat analysis using multi-parametric MRI.

**Methods:**

In this prospective study (NCT05868928), patients with brain metastases will undergo multi-parametric MRI before SRS, and then follow-up MRIs will be conducted every 3 months until 24 months after SRS. The multi-parametric MRI protocol will include T2-weighted and contrast-enhanced T1-weighted imaging, diffusion-weighted imaging, and dynamic susceptibility contrast imaging. Using k-means voxel-wise clustering, this study will define three structural MRI habitats (enhancing, solid low-enhancing, and nonviable) on T1- and T2-weighted images and three physiologic MRI habitats (hypervascular cellular, hypovascular cellular, and nonviable) on apparent diffusion coefficient maps and cerebral blood volume maps. Using RANO-BM criteria as the reference standard, via Cox proportional hazards analysis, the study will prospectively evaluate associations between parameters of the tumor habitats and the time to recurrence. The DICE similarity coefficients between the recurrence site and tumor habitats will be calculated.

**Discussion:**

The tumor habitat analysis will provide an objective and reliable measure for assessing tumor recurrence from brain metastasis following SRS. By identifying subregions for local recurrence, our study could guide the next therapeutic targets for patients after SRS.

**Trial registration:**

This study is registered at ClinicalTrials.gov (NCT05868928).

## Background

Stereotactic radiosurgery (SRS) has emerged as the standard of care for patients who have relatively few brain metastases (BMs) and do not require immediate relief of mass-related symptoms [1, 2]. However, challenges arise when an enlarging enhancing lesion is observed during follow-up magnetic resonance imaging (MRI) after SRS, as this lesion may indicate either tumor progression or radiation necrosis (RN). The incidence of RN following SRS is reported to range from 5 to 26% per treated lesion [3, 4]. Differentiating RN from tumor progression is crucial for patient management because the treatment strategies for these two conditions are substantially different. For recurrent tumors, repeat SRS or surgical resection may be considered, whereas additional radiotherapy should be avoided in cases of RN [5, 6].

On perfusion MRI, the relative cerebral blood volume (CBV) and cerebral blood flow (CBF) have shown pooled sensitivity and specificity values of around 85% and 81%, respectively, in distinguishing tumor progression from RN [7]. Nonetheless, the frequent coexistence of tumor recurrence and radiation injury in post-treatment tumors [8] and the heterogeneous nature of such tumors present significant challenges to the evaluation of post-treatment lesions using single quantitative parameters. A single parameter is only capable of providing information in one direction or a linear correlation, which limits the comprehensive characterization of post-treatment tumors. In this respect, pattern analysis combining quantitative parameters [9] may improve discrimination ability and tissue characterization in metastatic tumors after high-dose SRS.

Tumor habitat analysis aims to identify distinct subregions within a complex tumor by clustering similar voxels that share common tumor biology [10, 11]. A recent retrospective longitudinal study with diffusion-weighted and dynamic susceptibility contrast (DSC) imaging showed the feasibility of tumor habitat analysis, which reflects information on tumor cellularity and tumor vascularity [12]. This study demonstrated that an increase in hypovascular cellular habitat (low CBV and low apparent diffusion coefficient [ADC]) is predictive of early recurrence of BM after SRS.

For an imaging biomarker to be used as a generalizable tool, a prospective study is required to ensure that the biomarker provides a reliable quantitative assessment of treatment responses. Our study aims to validate an imaging risk for tumor recurrence after SRS on patients with BMs by conducting tumor habitat analysis using multi-parametric MRI.

## Methods

### Study objectives

#### Primary objective

This study aims to evaluate a multi-parametric MRI-based tumor habitat imaging biomarker for determining tumor recurrence after SRS for BMs. The primary outcome measure is the time to recurrence, calculated from the date of SRS for BMs until the date of progression assessed according to the Response Assessment in Neuro-Oncology Brain Metastases (RANO-BM) criteria. The timeframe will be restricted to up to 24 months after SRS.

#### Secondary objectives

1. To match the site of tumor recurrence with each tumor habitat.
2. To measure the per lesion–based response.
3. To evaluate the occurrence rate of RN, which will be determined through a combination of follow-up MRI findings and clinical evaluation by a multidisciplinary team.

### Study design

This prospective single-arm observational cohort study aims to investigate the value of multi-parametric MRI for predicting recurrence in patients with BMs who are treated with SRS. Patients will undergo the standard care protocol for their metastases. This study will not incorporate any interventions.

### Study procedures

**Fig. 1:**
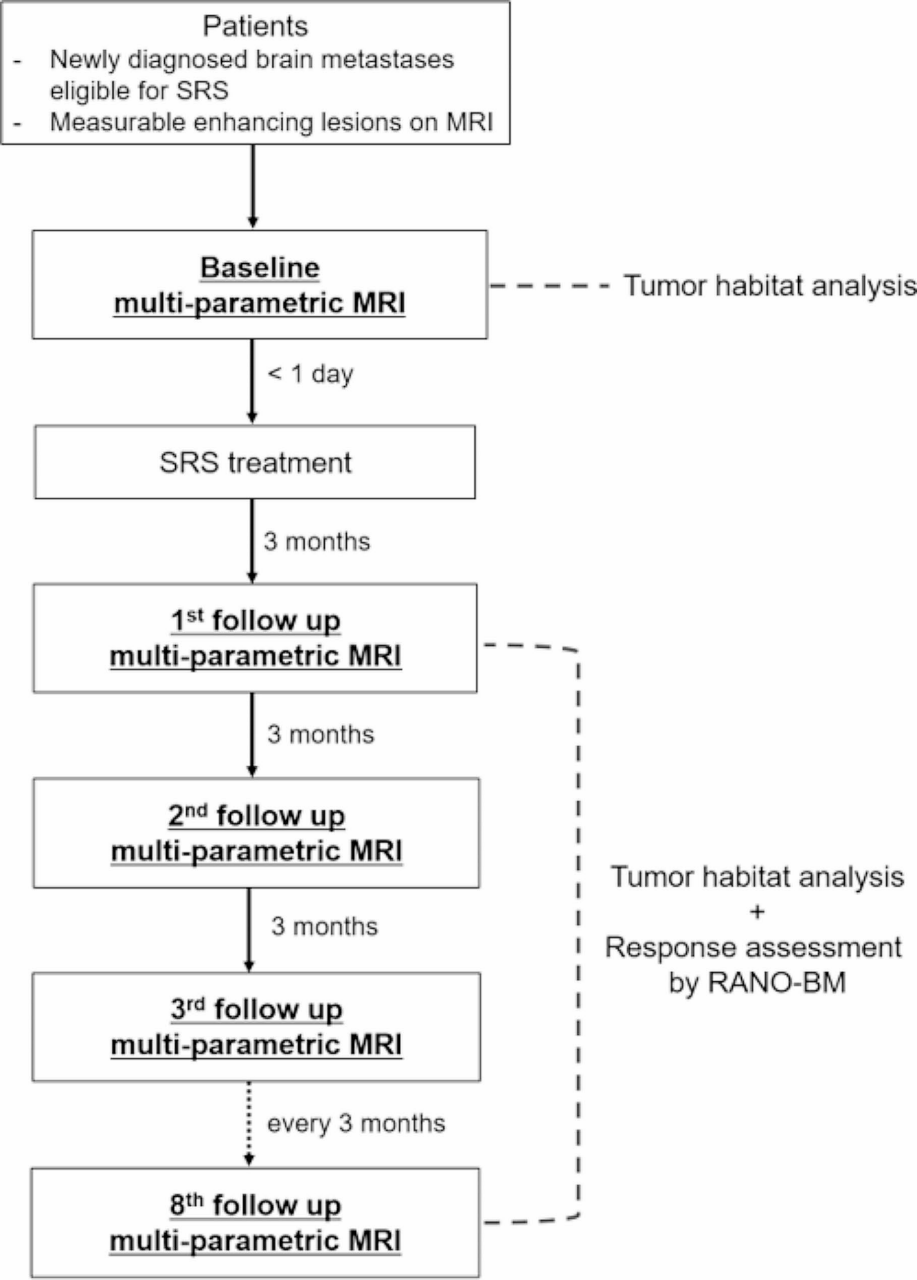
Study schema. SRS, stereotactic radiosurgery; RANO-BM, response assessment in neuro-oncology brain metastases

Figure 1 shows a flowchart of the study. Patients will undergo baseline MRI on either the day of SRS treatment or the previous day. After SRS treatment, follow-up MRI will be acquired every 3 months, up to 24 months after SRS.

### Patient selection

#### Inclusion criteria

1. Age ≥ 18 years.
2. Karnofsky Performance Status Scale score ≥ 70.
3. Brain MRI acquired within 1 month of enrollment.
4. Measurable enhancing lesions on MRI.
5. Undergoing SRS for BMs.
6. Patients with lesions eligible for SRS:

- One to ten newly diagnosed BMs.
- Patients without acute neurological symptoms.

7. A longest diameter > 1.5 cm for tumor habitat analysis.

#### Exclusion criteria

1. Age < 18 years.
2. Prior brain surgery, SRS, or whole-brain radiation therapy.
3. Diagnosis of leukemia, lymphoma, germ-cell tumor, small-cell lung cancer, leptomeningeal disease, or unknown primary tumor.
4. Without baseline MRI within 1 month of enrollment.
5. Non-measurable enhancing lesions on MRI: lesions with longest dimension < 10 mm, lesions with borders that cannot be reproducibly measured, dural metastases, bony skull metastases, and leptomeningeal disease.
6. Contraindications to MRI: electronic devices such as pacemakers or implantable defibrillators, metallic foreign bodies (e.g., intraocular metal), and severe claustrophobia.

### SRS treatment

Patients will receive standard SRS treatment as recommended by the neurosurgery team. Participating in this study will not alter the patient’s treatment course in any way.

### Image acquisition

All MRI scans will be acquired on a 3-T scanner (Ingenia 3.0 CX, Philips Healthcare). MRI will include conventional and advanced sequences consisting of pre- and contrast-enhanced (CE) 3D T1-weighted imaging (T1WI), T2-weighted imaging (T2WI), fluid-attenuated inversion recovery imaging (FLAIR), diffusion-weighted imaging (DWI), and dynamic susceptibility contrast (DSC) imaging.

The parameters for the T2WI will be as follows: repetition time (TR)/echo time (TE), 9000/135 ms; field of view (FOV), 240 × 240 mm; matrix, 256 × 256; and slice thickness, 4 mm. A parallel imaging technique will be used for the T2WI and FLAIR (SENSE; reduction factor = 2), CE-T1WI (CS-SENSE; reduction factor = 3.5), DWI (SENSE; reduction factor = 2), and DSC (SENSE; reduction factor = 3.2).

Gadolinium contrast agent (Gadoterate meglumine, Dotarem; Guerbet; 0.1 mmol/kg body weight) will be administered intravenously with a power injector (Spectris; Medrad). After starting IV contrast injection, a contrast-enhanced T1 image will be required with a delay of 2 min and 25 s.

#### DWI

The DWI parameters will be as follows: TR/TE, 3000/56 ms; diffusion gradient encoding, b = 0 and 1000 s/mm^2^; FOV, 250 × 250 mm; matrix, 256 × 256; and slice thickness/gap, 5/2 mm. ADC images will be calculated from b = 1000 and b = 0 s/mm^2^ images.

#### DSC imaging

DSC imaging will be acquired using a gradient-echo echo-planar imaging protocol. A preload of 0.01 mmol/kg gadoterate meglumine (Dotarem; Guerbet) will be administered, followed by a dynamic bolus of a standard dose of 0.1 mmol/kg gadoterate meglumine delivered at a rate of 4 mL/s by an MRI-compatible power injector (Spectris; Medrad). The contrast material bolus will be followed by injection of 20 mL of saline at the same injection rate.

The DSC imaging parameters will include the following: TR/TE, 1808/40 ms; flip angle, 35°˚; FOV, 240 × 240 mm; slice thickness/gap, 5/2 mm; matrix, 128 × 128; total acquisition time, 1 min and 54 s. The dynamic acquisition will be performed with a temporal resolution of 1.5 s, and 60 dynamic images will be acquired. DSC imaging will be acquired with complete tumor volume coverage and the same section orientation as that used for conventional MRI.

### Tumor habitat analysis

Tumor habitat analysis will be performed at baseline and for every follow-up MRI scan.

#### Deep learning segmentation and image processing

Brain extraction and deep learning segmentation will be performed on FLAIR and 3D CE-T1WI using an algorithm (https://github.com/MIC-DKFZ/nnUNet) in the PyTorch package (version 1.1) in Python 3.7. The segmentation of contrast-enhancing lesions, defined as the contrast-enhancing solid portions on CE-T1WI, will be performed and included in the tumor habitat analysis. Lesions showing similar high-signal intensity on both pre-contrast T1WI and CE-T1WI will be considered as hemorrhagic lesions and excluded from the analysis. This process will be supervised by an experienced neuroradiologist (J.E.P., with 10 years of experience).

The signal intensities of the T2WI and CE-T1WI will be normalized using the kernel density estimation–based white matter segmentation tool [13] in R (R Foundation for Statistical Computing, Vienna, Austria). A pharmacokinetic map will be computed using Nordic ICE software (NordicNeuroLab) for analysis of DSC. The blood quantity will be measured through an integrated DSC module, which combines a leakage correction algorithm for the relative CBV (rCBV) with manual noise thresholding. To generate normalized CBV (nCBV) maps, the rCBV maps will be adjusted to align them with the levels found in normal white matter.

To evaluate the changes on follow-up MRI, the 3D CE-T1WI of the patient will be co-registered and resampled into isometric voxels. The T2WI, nCBV, and ADC images will also be co-registered and resampled into the same space as the isovoxel CE-T1WI images using rigid transformations with six degrees of freedom in the SPM package (version 12; https://www.fil.ion.ucl.ac.uk/spm/).

#### Voxel clustering

**Fig. 2:**
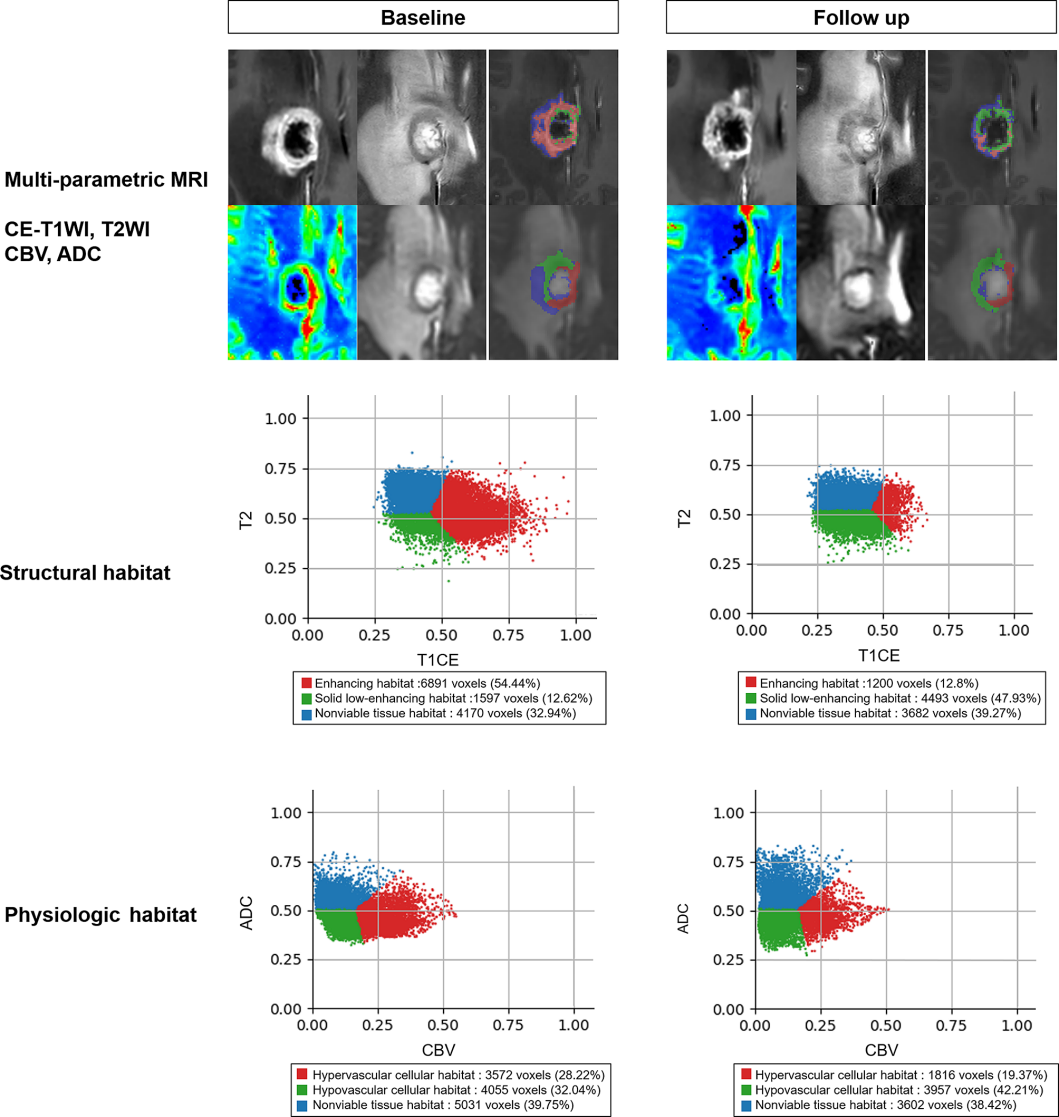
Example of tumor habitat analysis using multi-parametric MRI, as per the study protocol. To construct structural habitats, k-means clustering is applied to CE-T1WI and T2WI and the resulting habitats are color-coded as follows: enhancing habitats (red), solid low-enhancing habitats (green), and nonviable tissue habitats (blue). Similarly, for physiologic habitats, k-means clustering is applied to ADC and CBV images, and the representing habitats are color-coded as follows: hypervascular cellular habitats (red), hypovascular cellular habitats (green), and nonviable tissue habitats (blue). In this case, a patient was treated with stereotactic radiosurgery for brain metastasis in the right cingulate gyrus. Compared with the baseline MRI, the follow-up structural MRI analysis shows that the solid low-enhancing habitat increased (from 1597 voxels to 4493 voxels), whereas the other habitats decreased. In the physiologic MRI analysis, all habitats exhibited a decrease in voxel numbers, but the percentage of hypovascular cellular habitat increased from 32.04–42.21%. CE-T1WI, contrast-enhanced T1 weighted imaging; T2WI, T2-weighted imaging; ADC, apparent diffusion coefficient; CBV, cerebral blood volume

The voxel clusters will be aggregated on the basis of the T2WI and CE-T1WI signal intensities, or the nCBV and ADC values, using a k-means clustering algorithm in scikit-learn (https://github.com/scikit-learn/scikit-learn) in Python 3.7. Following the methods in previous studies, three clusters will be used for both the structural and physiologic MRI habitats [12, 14]. Figure 2 shows an example of tumor habitats that will be obtained using this study protocol.

#### Structural MRI habitats

The three clusters for structural MRI habitats will be defined using CE-T1WI and T2WI as follows: an “enhancing habitat” with high CE-T1 signal intensity irrespective of T2 signal intensity; a “solid low-enhancing habitat” with low T2 and CE-T1 signal intensity; and a “nonviable tissue habitat” with high T2 and low CE-T1 signal intensity.

#### Physiologic MRI habitats

The three clusters for physiologic MRI habitats will be defined using ADC and CBV maps as follows: a “hypervascular cellular habitat” with relatively low ADC and relatively high CBV values compared with other habitats; a “hypovascular cellular habitat” with relatively low ADC and relatively low CBV values; and a “nonviable tissue habitat” with relatively high ADC and relatively low CBV values.

### Reference standard

MRI analysis will be conducted by two researchers (Y.H.R. and N.K., with 6 and 4 years of experience in imaging analysis, respectively) as central reviewers. The readers will measure the SRS target BM lesions on baseline MR images and the corresponding lesions on follow-up MR images. The response assessment, comparing the baseline and follow-up images, will be determined using the RANO-BM criteria [15] on per-lesion and per-patient bases.

#### Complete response

A complete response is defined as the disappearance of all target lesions with no new lesions, no use of corticosteroids, and the patient in a stable or clinically improved condition.

#### Partial response

A partial response is defined as at least a 30% decrease in the sum of the longest diameters of the target lesions, taking as reference the baseline sum of the longest diameters, no new lesions, stable to decreased corticosteroid dosage, and the patient in a stable or clinically improved condition.

#### Stable disease

Stable disease will be defined when there is neither sufficient shrinkage to qualify for partial response nor sufficient growth to qualify for progressive disease, taking as reference the smallest sum of the longest diameters of the patient’s tumors while they are enrolled in the study.

#### Progressive disease

Progressive disease is defined as at least a 20% increase in the sum of the longest diameters of the target lesions, taking as reference the smallest sum in the study (this includes the baseline sum if that is the smallest in the study). In addition to a relative increase of 20%, at least one lesion must grow by an absolute value of 5 mm or more to be considered as progression. When there are mixed signs of progression and RN, the response assessment will continue to adhere to the RANO-BM criteria, as described. If lesions grow according to the definition of “progressive disease,” they will be classified accordingly.

#### RN

The reference standard of RN or tumor progression will be diagnosed according to the clinicoradiological consensus of a multidisciplinary team including two neuroradiologists (J.E.P. and H.S.K., with 10 and 25 years of experience in neuro-oncologic imaging, respectively), two neurosurgeons (Y.H.K. and Y.H.C., with 20 years and 23 years of experience, respectively) and a neuro-oncologist (S.K.Y. with 13 years of experience). The multidisciplinary team will review all imaging and medical records. RN will be diagnosed when there is an increase in the contrast-enhancing lesion size and it subsequently regresses or becomes stable without any change in treatment at least 6 months after SRS, and when there are progressive neurological signs corresponding to the location of the RN [16, 17]. Additional imaging modalities, such as positron emission tomography or single-photon emission tomography, may be used along with MRI if deemed clinically necessary and will be considered during the multidisciplinary discussions for diagnosing RN.

Additionally, when such a lesion yields a symptomatic mass effect, a second-look operation is justified to confirm the pathological diagnosis. Expert neuropathologists in our hospital will review the lesion specimens to determine whether the findings indicate RN or tumor progression when surgical resection is performed. RN will be diagnosed as described in previous literature, characterized by an area of necrosis that appears hypocellular and is sharply demarcated from the surrounding gliotic brain, with necrotic, hyalinized blood vessels [18–20]. When histologic features of both RN and tumor progression are present in specimens, those composed of more than 25% tumor cells will be defined as recurrent tumors, whereas those with less than 25% tumor cells will be classified as RN [21].

### Sample size calculation

The sample size for this prospective study was determined according to binomial receiver operating characteristic curve analysis performed in diagnostic accuracy studies by a biostatistician (S.Y.P., with 12 years of experience). The sample size was calculated using PASS 15 Power Analysis and Sample Size (NCSS, Kaysville, Utah, ncss.com/software/pass). The null hypothesis of an area under the curve (AUC) of 0.7 was compared with an alternative hypothesis of an AUC of 0.85 for the tumor habitat analysis for the determination of RN. The target distribution was the rate of RN versus tumor recurrence, which is known to be detected in a 1:2 ratio in post-treatment BM [4]. With an alpha of 0.05 (type-1 error) and a beta of 0.20 (type-2 error), 34 positive cases (RN) and 68 negative cases (tumor recurrence), giving a total of 102 cases, were identified as the appropriate sample size. Considering a dropout rate of 10% and a censoring rate of 20% for BM, 132 patients were found to be appropriate for the study design.

### Statistical analysis

Analysis of the association between the tumor habitats and time to recurrence will be conducted using Cox proportional hazards regression analysis. The imaging predictor exhibiting a strong association will be selected on the basis of both univariate and multivariable Cox proportional regression analysis. Adjustments for time-dependent covariates will be made.

The prediction model for time to recurrence will be created by combining imaging predictors and clinical variables. The performance of the prediction model will be analyzed using C-statistics and time-dependent receiver operating characteristic curve analysis, and calibration (Hosmer-Lemeshow goodness-of-fit test) will be evaluated.

The Dice similarity coefficient will be calculated when the recurrence is diagnosed. The area of each habitat before SRS will be divided by the contrast-enhancing lesion at the time of tumor recurrence.

## Discussion

This study aims to prospectively evaluate a tumor habitat-based imaging biomarker to predict tumor recurrence in SRS-treated BM. Tumor habitat analysis will be conducted using baseline multi-parametric MR images and follow-up MR images captured after SRS treatment. Metastatic tumors are biologically complex and exhibit significant spatial variation in their structural properties. This intratumoral and peritumoral heterogeneity becomes even more complex after SRS, when it becomes combined with radiation-induced changes. The grouping of voxels of similar signal intensity may enable identification of subregions that share common biologic characteristics and respond differently to treatment, providing information that may not be detectable through visual analysis.

A previous autopsy study indicated that the enhancing areas of post-SRS lesions consist of various pathologic components, including viable tumor tissue, necrotic tissue, inflammatory cells, and vessels [22]. The degree of enhancement cannot differentiate between these complex components, and analyses relying solely on enhancing lesions cannot distinguish progression and radiation-induced changes. Previous studies have demonstrated the predictive value of the correspondence or proportion of solid lesions on T2WI and enhancing lesions on CE-T1WI for diagnosing viable tumors [23, 24]. Interval decreases in the ADC or rCBV values of treated lesions have also been reported to indicate tumor responses following SRS [25–28]. However, visual assessment of structural MRI is limited in terms of reproducibility and its inherent subjectivity. Furthermore, a single quantitative parameter may fail to provide a thorough explanation of the intricate tumor biology mixed with RN. Thus, in this study, we will use CE-T1WI and T2WI for the structural tumor habitat analysis, and ADC and CBV maps for the physiologic tumor habitat analysis. The prospective longitudinal analysis of tumor habitats will enable us to track changes across different subregions in fine detail.

The imaging biomarker developed in this prospective study will provide an objective and reliable measure for assessing tumor recurrence following SRS. Identification of the subregions at increased risk of recurrence will facilitate the establishment of an optimal strategy for close monitoring of therapeutic targets and distinguishing nonviable tumor areas that can be considered safe for observation. Such an accurate imaging assessment of tumor progression or radiation-induced change will help avoid unnecessary surgery or radiation therapy, thereby minimizing associated patient morbidity.

## Data Availability

Not applicable.

## Abbreviations

SRS: Streotactic radiosurgery
BM: Brain metastasis
RN: Radiation necrosis
CBV: Cerebral blood volume
CBF: Cerebral blood flow
DSC: Dynamic susceptibility contrast
ADC: Apparent diffusion coefficient
RANO-BM: Response Assessment in Neuro-Oncology Brain Metastases
